# The role of tanycytes in hypothalamic glucosensing

**DOI:** 10.1111/jcmm.12590

**Published:** 2015-06-17

**Authors:** Roberto Elizondo-Vega, Christian Cortes-Campos, Maria J Barahona, Karina A Oyarce, Claudio A Carril, Maria A García-Robles

**Affiliations:** a Laboratorio de Biología Celular, Departamento de Biología Celular, Facultad de Ciencias Biológicas, Universidad de ConcepciónConcepción, Chile; b Whitehead Institute for Biomedical ResearchCambridge, MA, USA

**Keywords:** monocarboxylate transporters, glucose transporters, glucokinase, lactate, feeding behaviour, tanycytes, hypothalamus, glucosensing

## Abstract

Tanycytes are elongated hypothalamic glial cells that cover the basal walls of the third ventricle; their apical regions contact the cerebrospinal fluid (CSF), and their processes reach hypothalamic neuronal nuclei that control the energy status of an organism. These nuclei maintain the balance between energy expenditure and intake, integrating several peripheral signals and triggering cellular responses that modify the feeding behaviour and peripheral glucose homeostasis. One of the most important and well-studied signals that control this process is glucose; however, the mechanism by which this molecule is sensed remains unknown. We along with others have proposed that tanycytes play a key role in this process, transducing changes in CSF glucose concentration to the neurons that control energy status. Recent studies have demonstrated the expression and function of monocarboxylate transporters and canonical pancreatic β cell glucose sensing molecules, including glucose transporter 2 and glucokinase, in tanycytes. These and other data, which will be discussed in this review, suggest that hypothalamic glucosensing is mediated through a metabolic interaction between tanycytes and neurons through lactate. This article will summarize the recent evidence that supports the importance of tanycytes in hypothalamic glucosensing, and discuss the possible mechanisms involved in this process. Finally, it is important to highlight that a detailed analysis of this mechanism could represent an opportunity to understand the evolution of associated pathologies, including diabetes and obesity, and identify new candidates for therapeutic intervention.

- Introduction
- Morphological characteristics of the hypothalamic region
- Hypothalamic tanycytes
- Hypothalamic glucosensing
- Neuronal-glial interaction in the hypothalamic glucosensing
- MCTs and their participation in the cerebral glucosensing mechanism
- The role of glucose transporters in hypothalamic glucosensing
- Participation of GK in hypothalamic glucosensing
- Conclusions

## Introduction

Control of feeding behaviour and glucose homeostasis relies on the cerebral capacity to integrate diverse peripheral signals, including leptin, insulin, glucagon, ghrelin and glucose, that reflect the nutritional and energetic state of the organism, as well as its ability to generate responses that can regulate feeding behaviour, energy expenditure and the metabolic activity of cells 1–5. For several decades, it has been known that brain function is glucose-dependent 6, and that glucose modulates feeding behaviour 7. In 1919, Anton Carlson suggested that low plasma glucose concentrations could be a signal for meal initiation and high glucose concentration could result in meal termination 7. Subsequent studies have shown that lesions in specifics areas of the brain, such as the ventromedial 8–10 and lateral hypothalamus (LH) 11 affect feeding behaviour. These findings led Mayer in 1953 to propose the glucostatic hypothesis, which establish a connection between blood glucose concentrations and appetite 12. This hypothesis indicates that rises in plasma glucose concentration after a meal are sensed by hypothalamic neurons that respond by triggering meal termination 12. Currently, exist a great interest in understand the precise molecular and cellular mechanism that control the glucosensing. Given diseases such as diabetes and obesity can be induced by a deregulation in this process.

Two different populations of glucose responsive neurons have been identified in the hypothalamus. Neurons that increase their firing rate and neurons that decrease their firing rate in response to rises of glucose. These neurons are located in the ventromedial hypothalamus (VMH) and the LH 13. However, physiological glucose concentrations in the brain parenchyma (1.4 mM in normoglycaemic rats and 3.3 mM in hyperglycaemic rats) never reach the concentrations used in most studies to identify these glucose-responsive populations and demonstrate their changes in firing rate 14–17. Therefore, the effect of physiological glucose concentration over the activity of the hypothalamic glucosensing neurons remains a matter of debate 18,19. However, a proportional relationship between glucose levels in blood and the cerebrospinal fluid (CSF) has been reported 20–22. The CSF is the only fluid in the brain, in which significant changes in glucose concentration have been detected during hyperglycaemia, reaching levels as high as 15 mM 23,24. Moreover, analysis of the hypothalamic cytoarchitecture indicates that the nuclei involved in glucose homeostasis are not in direct contact with the CSF; however, hypothalamic ependymal cells (*i.e*. tanycytes), that cover the ventricular walls, make contact with both the CSF and neuronal nuclei that control the feeding behaviour 25. These background data, led us to propose that hypothalamic tanycytes are responsible, at least in part, for sensing changes in glucose levels in the CSF and transduce this signal to neighbouring neurons, triggering a response in these cells. In this review, we focus on information that supports tanycytic glucosensing and possible mechanisms involved in this process.

## Morphological characteristics of the hypothalamic region

**Figure 1:**
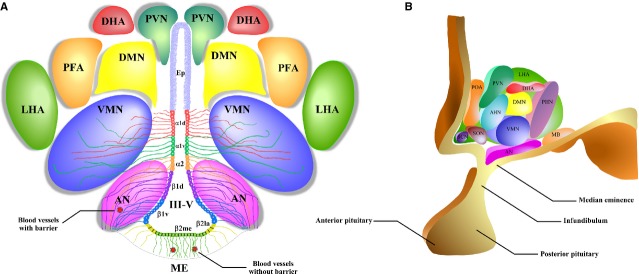
A schematic representation of the hypothalamic nuclei and the distribution of tanycytes over the wall of the third ventricle (III-V). (A) Coronal view of the approximate location of the hypothalamic nuclei and tanycytes. Ciliated ependymocytes (ep) line the dorsal wall of the III-V. The α1d-tanycytes (α1d) and α1v-tanycytes (α1v) have long projections that make contact with the neurons of the VMN. α2-tancycytes (α2) have projections to the AN and blood vessels. In a more ventral section of the III-V, the β1d-tanycytes (β1d) and β1v-tanycytes (β1v) make projections to the AN, making contact with orexigenic and anorexigenic neurons and blood vessels. In the floor of the III-V, the β2la-tanycytes (β2la) and β2me-tanycytes (β2me) are joined by tight junctions forming part of the median eminence (ME)-cerebrospinal fluid (CSF) barrier, and their projections make contact with the fenestrated blood vessels of the ME. (B) Sagittal view of the distribution of the hypothalamic nuclei. Ep: ependymocytes; AN: arcuate nucleus; VMN: ventromedial nucleus; DMN: dorsomedial nucleus; PVN: periventricular nucleus; DHA: dorsal hypothalamic area; PFA: perifornical area; LHA: lateral hypothalamic area; SCN: suprachiasmatic nucleus; SON: supraoptic nucleus; POA: preoptic area; MB: mammillary bodies; ME: median eminence; III-V: third ventricle.

The hypothalamus can be divided into three zones: (*i*) the periventricular zone formed by the preoptic area (POA), suprachiasmatic nucleus (SCN), paraventricular nucleus (PVN), arcuate nucleus (AN) and the posterior nucleus; (*ii*) the medial zone formed by the medial PON, anterior hypothalamic nucleus (AHN), ventromedial nucleus (VMN), dorsomedial nucleus (DMN) and premammillary nucleus and (*iii*) the lateral hypothalamic area (LHA) formed by the lateral preoptic nucleus, lateral hypothalamic nucleus, tuberomammillary nucleus and supraoptic nucleus (Fig.1A and B) 26. The median eminence (ME) is located in the middle-basal hypothalamus and dorsal side borders the infundibular recess of the third ventricle (III-V), making contact with the CSF. Its ventral side borders the pars tuberalis of the pituitary, making contact with the perivascular space of the portal capillary system and the subarachnoid CSF 27. The ependymal region of ME is formed by β2-tanycytes with tight junctions that form a barrier between the ME and the ventricular CSF, known as the CSF-ME barrier 27–29. However, the blood vessels of the ME do not form a blood-brain barrier (BBB); thus, the ME is a circumventricular organ, known to be ‘windows to the brain’. It has been recently reported that the nutritional status of an individual modulates the permeability of ME to circulation metabolic signals 30,31. Further studies are needed to show whether metabolic signals are transferred directly from the blood to AN neurons or transferred from fenestrated vessels to the processes of β2-tanycytes, and subsequently CSF to quickly generate an increase in glucose concentration at the infundibular recess area proportional to that in blood (β1-tanycytes in contact with AN neurons).

## Hypothalamic tanycytes

A tanycyte is a specialized type of ependymal cell, localized in the lower parts of the ventricular walls and the floor of the III-V. Tanycytes have an elongated morphology and are not ciliated 32, and they are classified based on their distribution in the hypothalamic ventricular wall 33. Basal processes of α1-tanycytes project towards the VMN, while those of α2-tanycytes project to the AN, forming an interphase between the CSF and the neuronal nuclei that allows the exchange of molecules 34. β1-tanycytes line the infundibular recess, and their basal projections reach the lateral regions of the ME and the AN. β2-tanycytes cover the floor of the III-V and extend their projections inside the ME.

To better understand the role of each population of tanycytes, morphological studies and expression analysis of molecular markers have identified the following subpopulations: dorsal and ventral α1-tanycytes (α1d and α1v), α2-tanycytes, dorsal and ventral β1-tanycytes (β1d and β1v) and lateral (β2la) and medial β2-tanycytes (β2me) 35–37 (Fig.1A). The ventricular wall, which is comprised of α and β1-tanycytes in the ventral region of the hypothalamus, contains few subependymal astrocytes, indicating that tanycytes are the main glial cell type present in this area 34,38. Moreover, β2-tanycytes lying on the ME have privileged access, *via* fenestrated capillaries 39, to nutritional signals carried by the bloodstream, such as glucose and hormones 40,41. Furthermore, the basal processes of tanycytes penetrate into the hypothalamic parenchyma, contacting AN neurons that participate in the regulation of food intake (FI) 25,33.

## Hypothalamic glucosensing

Supporting the glucostatic hypothesis, *in vivo* studies have demonstrated destroying selected hypothalamic nuclei or regions, including the VMH, induces hyperphagia and obesity, while the ablation of the LHA, leads to hypophagia and loss of bodyweight 42,43. Electrophysiological studies in brain slices have demonstrated the presence of hypothalamic neurons that can increase or reduce the frequency of their electric activity as a function of increased glucose 44 and lactate 45 concentrations and have been classified as glucose-exited (GE) and glucose-inhibited (GI) neurons, respectively 13,46,47, located in the AN, VMN, PVN and LHA 48–50.

Patch clamp recordings in mouse brain slices incubated with an extracellular medium containing d-glucose have led to propose the existence of two more neuronal populations: high glucose-excited and high glucose-inhibited neurons 51,52. These studies indicate that neurons can be directly or indirectly activated or inhibited by glucose, and this metabolic substrate is not solely used as metabolic substrate, but also as signalling molecules that correspond with the energetic status of the organism, allowing the release of hormones, neurotransmitters and/or neuropeptides that control FI 53.

The AN has a central role in the integration of hormonal, nutritional and neuronal signals derived from peripheral organs. For example, the AN responds to peripheral signals, such as leptin and ghrelin, and further controls secondary neuronal populations in the PVN, DMN and LHA, which process information regarding energy homeostasis 54–56. The AN is composed of neuronal populations with antagonistic functions, including neurons that inhibit FI through the release of anorexigenic peptides (α-melanocyte-stimulating hormone [α-MSH], a processing product of pro-opiomelanocortin (POMC) and the cocaine- and amphetamine-regulated transcript) 54,57 as well as those capable of stimulating FI through the secretion of orexigenic peptides (neuropetide Y [NPY] and the agouti-related peptide) 58,59. Studies in brain slices showed that 40% of NPY neurons are GI neurons 60, but the identity of GE neurons is not completely clear and could correspond to POMC-positive neurons 61,62. This directly correlates with changes in neuronal activity induced by variations in glucose concentration related with the control of FI. *In vivo* studies showed that lateral intracerebroventricular (i.c.v.) injection of glucose in mice mimics hyperglycaemia at 2 hrs after the injection, as detected by reduced NPY and increased POMC mRNA levels, which was correlated with the cessation of FI 63,64. Since AN neurons are not in direct contact with blood or CSF 25,33,35,36,38,65, an alternative pathway has been proposed, which involves a metabolic interaction between AN neurons and tanycytes *via* lactate 25,34–36,38,66. *In situ* studies using patch clamp analysis and single-cell extracellular recordings in brain slices of rats have shown that lactate can increase the action potential frequency, of GE neurons from the VMH 45,67, suggesting that this monocarboxylate is required for glucosensing in the brain. Similarly, *in vivo* studies have demonstrated that i.c.v. lactate injections into the III-V decrease blood glucose levels response that is disrupted when lactate or glucose is co-injected with oxamate, an inhibitor of the lactic dehydrogenase enzyme, confirming that lactate uptake in the hypothalamus is essential for glucose homeostasis 68. Similarly, lactate injection through the carotid artery in rats led a transient increase in insulin secretion 69.

## Neuronal-glial interaction in the hypothalamic glucosensing

In the brain, neurons have higher energy requirements than glia, but different reports show that glucose metabolism is slower in neurons in cultures or that found in brain slices than glial cells under similar conditions 70–74. In addition, neurons do not have direct access to glucose due to the presence of the BBB. Thus, nutrients need to pass across the BBB, and this diffusion is driven by the concentration gradient between the blood and the interstitial fluid through the glucose transporter 1 (GLUT1) 18,75. An alternative scenario has been proposed in which neurons use a substrate other than glucose to supply their energetic demands, which is known as the astrocyte-neuron lactate shuttle hypothesis 76. Several studies support the hypothesis of a functional coupling between glia and neurons mediated by lactate, for instance in peripheral sensory organs, such as the retina, an interaction between Müller cells and photoreceptor neurons has been shown 77, and in olfactory epithelia metabolic coupling between olfactory neurons and their supporting glial cells, has been proposed 78. *In vivo* studies have demonstrated that alterations induced by insulin-induced hypoglycaemia are completely prevented by lactate infusion and that the brain oxidizes lactate in an activity-dependent manner, suggesting that the brain prefers lactate over glucose even in the presence of both substrates 79. Moreover, interruption of lactate uptake in the hippocampus generates amnesia that can be rescued by lactate but not with glucose, showing lactate is essential for the establishment long-term memory formation 80. Therefore, we and other investigators have proposed that hypothalamic glucosensing is mediated by a metabolic interaction between glial cells (*i.e*. astrocytes and tanycytes) and neuroendocrine neurons that control the feeding behaviour 25,36,66,81,82. The important metabolic, structural and homeostatic functions of astrocytes have been extensively reviewed 83,84.

Glucose-exited neurons increase their electrical activity in response to glucose through a mechanism similar to that of glucose-induced insulin release in pancreatic β cells 19. In response to increased glucose concentrations, neurons increase their cytosolic ATP concentration ([ATP]c), which inhibits K_ATP_ channels and induces a change in membrane potential that, in turn, triggers the opening of voltage-gated Ca^2+^ channels and the subsequent uptake of Ca^2+^ and release of neurotransmitters, including neuropeptides 85. Using primary cultures of hypothalamic neurons and glial cells, dynamic bioluminescence imaging analysis, which records [ATP]c in real-time, revealed that glucose concentrations from 3 to 15 mM do not increase [ATP]c to induce closure of K_ATP_ channels and the consequent neuronal depolarization 70. However, exposure of hypothalamic neurons to 5 mM lactate (but not pyruvate) increased the amount of [ATP]c (in a oligomycin-sensitive way) enough to generate the closing of K_ATP_ channels. Exposure of primary cultures of hypothalamic glial cells to extracellular glucose concentration ranging from 3 to 15 mM significantly increased [ATP]c, which was not observed with lactate 70. These results suggest that lactate released from neighbouring glial cells could activate hypothalamic GE neurons in high glucose conditions. The participation of lactate in the glucosensing mechanism and feeding behaviour is supported by *in vivo* studies, in which i.c.v. injection of lactate into the III-V of the hypothalamus mimic the effect of hypothalamic glucose administration, generating lower FI and a reduction of bodyweight 86.

Moreover, in primary cultures of tanycytes, that elevation of extracellular glucose (from 2 to 10 mM) induced a rise in intracellular free Ca^2+^ concentration, which was dependent upon ATP generated by glycolysis and subsequent release through hemichannels formed by connexin 43 (HC-Cx43), but not by oxidative metabolism 87. *In situ* analysis in brain slices has shown that an acute application of glucose or non-metabolizable analogs of glucose over tanycyte cell bodies evoked robust ATP-mediated Ca^2+^ responses 88, suggesting that the pancreatic β cell paradigm does not apply to these cells. However, these studies showed that Ca^2+^ waves that depend on intracellular stores) were dependent on ATP release and P2Y receptor activation 87. Thus, tanycytes may sense glucose by more than one mechanism, which is determined by the subpopulation of tanycytes. Both *in vitro* and *in situ* studies demonstrated that tanycytes sense and respond to extracellular glucose *via* a rapid, glucose-activated signal transduction pathway mediated by lactate and/or ATP. Future *in vivo* studies will be required to determine whether tanycytes could sense extracellular changes in glucose concentration and transmit them to neurons *via* Ca^2+^ waves and/or the release of paracrine factors (*e.g*. ATP).

## MCTs and their participation in the cerebral glucosensing mechanism

**Figure 2:**
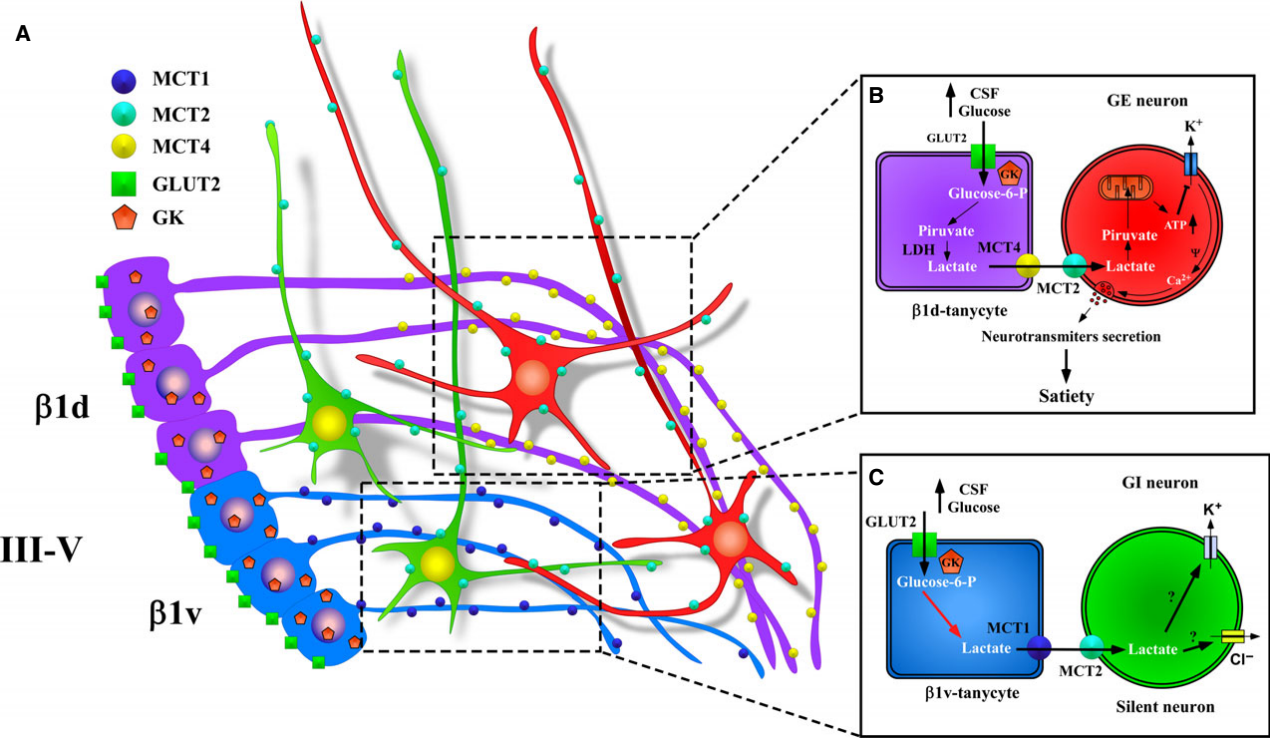
Model of cerebral glucose sensing based on the metabolic interaction between β1d-tanycytes or β1v-tanycytes and neurons. (A) Schematic representation of the location of MCT4 (yellow) in β1d-tanycytes processes (purple), MCT1 (blue) in β1v-tanycytes processes (light blue), and MCT2 (light green) in orexigenic (green) or GI neurons and anorexigenic (red) or GE neurons of the AN. (B) Schematic overview of the classical model of glial-neuronal interaction based on the transfer of lactate proposed for cerebral glucose sensing between GE neurons and tanycytes. (C) Scheme based on proposed interaction between β1v-tanycytes and GI neurons (orexigenic) compared to the increase in glucose concentration in the CSF. III-V: third ventricle; β1d and β1v: tanycytes; GE: glucose-excited neurons; GI: glucose-inhibited neurons, CSF: cerebral spinal fluid; GK: glucokinase; LDH: lactate dehydrogenase.

The monocarboxylate transporter (MCT) family is formed by 14 isoforms (MCT1-14), which use an electrochemical proton gradient to translocate monocarboxylates (*e.g*. l-acetate, l-acetoacetate and DL-β-hydroxybutyrate), in a stoichiometrical relationship of 1:1. Only MCT1-MCT4 have been demonstrated by functional characterization to be true MCTs; MCT8 is really a thyroid hormones transporter (Table1). The expression and distribution of MCTs have been recently reviewed 89,90. Here, we focus on their localization and relevance in the hypothalamus. MCT1 is expressed in lactate-producing (*e.g*. erythrocytes) and lactate consuming tissues (*e.g*. heart) 89,91. Monocarboxylate transporter 4 has been observed in lactate producing tissues (*e.g*. skeletal muscle and astrocytes) 92,93. In contrast, MCT2 is expressed in cell types, which use lactate, and is mainly restricted to neurons of different brain regions 94. Monocarboxylate transporter 3 has not been reported in hypothalamus. The expression of MCTs in the hypothalamus has been evaluated in only a few reports. Monocarboxylate transporter 1 was first detected in primary cultures of hypothalamic neurons and glia by immunostaining 70. Monocarboxylate transporter 4 was immunolocalized to some astrocytes and ciliated ependymal cells of the PVN 95, and MCT2 expression was detected in some neurons in the AN, DMH and the AHN in rats that consumed a high fat diet 96. A more detailed study indicates that MCT1 is present in the endothelial cells and α and β-tanycytes that line the ventricular walls and the floor of the III-V 36. In α-tanycytes, MCT1 is polarized in the ventricular cellular membranes and end-feet processes contacting the endothelial cells of the blood vessels 36. In β1v-tanycytes MCT1 is polarized to the apical membrane and cellular processes that contact neurons from the AN (orexigenic area), blood vessels and the external region of the brain 36. Monocarboxylate transporter 4 is also expressed in the hypothalamus, but it is mainly located in the lateral region of the AN (an anorexigenic zone), particularly in processes of β1d-tanycytes. Moreover, MCT1 and MCT4 function have been corroborated by *in vitro* studies using primary cultures of tanycytes and uptake of radiolabeled lactate. Additionally, we demonstrated that tanycytes release lactate in the presence of 5 mM glucose through MCT1 and MCT4 36. Furthermore, the coincident expression of MCT2 in orexigenic neurons 35 and MCT1 in β1v-tanycytes 36 (Fig.2A) led us to propose that these glial cells regulate the activity of GI neurons, and that lactate may inhibit these neurons, causing hyperpolarization *via* opening of Cl^−^ and/or K^+^ channels 51 (Fig.2C). Moreover, the localization of MCT4 in β1d-tanycytes 36 that contact GE POMC-reactive neurons MCT2 positives 35 suggests that these cells could be metabolically coupled through lactate (Fig.2A). The lactate released through MCT4 and incorporated by neurons through MCT2 could increase ATP levels, causing closure of K^+^ channels sensitive to ATP and increased neuronal electrical activity 70 (Fig.2B). This is also supported by GE neurons in the VMH and NTS that respond to increase lactate concentrations 45,97. Therefore, it is feasible that lactate has a dual role in the control of feeding behaviour, which is dependent upon the subtype of neuronal and glial cells activated in the process.

**Table 1:** Km values of MCT isoforms expressed in brain and their kinetic characterization

Protein/Gene names	Substrates	Km for lactate (mM)	Km for pyruvate (mM)	Km for d-β-hydroxy-butyrate (mM)	Km for Acetoa-cetate (mM)	Expression in brain	References
MCT1/SLC16A1	Lactate; Pyruvate; Ketones bodies	7.7	1.0	12.5	5.5	Cortical, hippocampal and supraoptic nucleus astrocytes; Choroid plexus; ciliated ependymal cells; endothelial cells; pericytes; α and β-tanycytes; oligodendrocytes; activated microglial cells; some populations of hypothalamic neurons	36,70,94,155–163
MCT2/SLC16A7	Lactate; Pyruvate; Ketones bodies	0.74	0.08	1.2	0.8	Neurons of cerebral cortex; Purkinje cells; ependymal cells; subependymal astrocytes of hypothalamus; orexigenic and anorexigenic neurons of hypothalamus	35,156,159,164–167
MCT3/SLC16A8	Lactate	5.8	_	_	_	Choroid plexus basolateral membrane	168,169
MCT4/SLC16A3	Lactate; Pyruvate; Ketones bodies	34	153	64	31	Bergmann glia; cerebellum, hippocampus and corpus callosum astrocytes; cerebral cortex; ciliated ependymal cells; α and β-tanycytes	36,93,94,155–157,164,170
MCT8/SLC16A2	T2; T3; rT3; T4	_	_	_	_	Choroid plexus; amygdala; hippocampus; olfactory bulb; hypothalamus	171–173

## The role of glucose transporters in hypothalamic glucosensing

Two families of transmembrane transporters mediate the membrane transport of glucose: the facilitative hexose transporters, GLUTs 98,99, and the sodium-glucose linked transporters (SGLTs) 100. Because the expression and distribution of GLUTs and SGLTs have been extensively reviewed elsewhere 99,101,102, we will focus on their expression and relevance in the hypothalamic glucosensing.

*In vitro* analyses detected expression of SGLT1, SGLT3a and SGLT3b in cultured neurons and adult rat hypothalamus 103, but *in vivo* studies have only shown SGLT1 expression in the PVN 104. Moreover, *in vitro* functional studies showed that 67% of GE hypothalamic neurons are activated by α-methylglucopyranoside, a non-metabolizable substrate of SGLT, and this effect was abolished by phloridzin (SGLT antagonist) 103. A possible non-metabolic glucose sensing mechanism in the hypothalamus has been propose, which involves GE neuronal activation in response to high glucose generated by the inward current triggered by co-transport of two sodium ions and glucose through SGLTs 105–107. Supporting experiments showed that i.c.v. administration of phloridzin enhances FI in rats 107 and inhibits glucose-induced activation of GE neurons in the VMH 45. Therefore, the role of SGLTs in hypothalamic glucose sensing needs to be examined in more depth, in particular to define the sub-population of GE neurons that express it and the physiological importance of this non-metabolic glucose sensing mechanism in feeding behaviour.

Glucose transporter 1 and GLUT3 are the predominant GLUT isoforms expressed in the brain, and are localized mainly in glia and neurons, respectively 108–110. In the hypothalamus, immunohistochemistry analysis revealed GLUT1 expression in glial and endothelial cells of the BBB in the VMH; however, it was not observed in neuronal cells 111,112. Immunocytochemistry and *in situ* hybridization also showed that GLUT1 is highly expressed in α and β1-tanycytes, with intense immunoreaction in cell processes located throughout the AN and in cell processes contacting the hypothalamic capillaries 34,113. Under normoglycaemic conditions, glucose levels in the brain are similar to the Km value of GLUT1 (Km = 1–5 mM) 114,115. Thus, the normal supply of energy to the brain is not rate limiting; however, several studies indicate that the energetic metabolism of glucose is limited by the capacity to phosphorylate the incorporated glucose by hexokinases 116,117.

Glucose transporter 3 has an elevated affinity for glucose with a reported Km of 1.4 mM 118. Despite its high glucose affinity, which normally implies a low transport capacity at high glucose levels, the activity of this transporter is dependent on its catalytic constant or Kcat, which is eightfold higher than astrocytic GLUT1 119. Therefore, it is possible that neurons expressing GLUT3 could respond to high glucose levels 120. Within the hypothalamus, immunohistochemical localization of GLUT3 was detected in neurons of the LHA, DMN and PVN 112. Although single-cell RT-PCR analysis revealed that GLUT3 as well as GLUT4 and GLUT2 are expressed in GI and GE neurons of the VMN 121, it is important to mention that mRNA may not directly reflect the amount, location or expression of these proteins; thus, it remains necessary to demonstrate their protein expression.

Glucose transporter 2 is a low-affinity/high-capacity transporter for glucose with a reported Km of 17 mM 25,101,122,123. Its association with the glycolytic enzyme, glucokinase (GK), allows an efficient uptake capacity at high glucose concentrations, which make GLUT2 and GK the ideal molecules that define a glucose sensor 124. Glucose transporter 2 mRNA was detected by *in situ* hybridization in human hypothalamic tissues in the VMN and AN 125. qRT-PCR analysis and genetic reporter (eYFP mice) studies indicate that GLUT2 is expressed in the LHA, VMH and DMH 126,127; however, it is not expressed in neuronal bodies of the AN. NPY and POMC neurons were, however, connected to nerve terminals positive for GLUT2; astrocytes and ependymocytes were also GLUT2-positive 127. Contradictory results have been reported using conventional and electron microscopy immunocytochemical analysis, which indicate that GLUT2 is localized in the neuronal cell bodies of the AN and corroborate the expression of GLUT2 in nerve terminals, astrocytes and ependymocytes near the III-V 128,129. Divergent results might be explained by the different methodologies employed. However, both *in situ* hybridization and immunocytochemical analyses have shown that GLUT2 is expressed in ependymal cells, specifically in the apical ventricular membranes of β1 and β2- tanycytes and was absent from neurons, endothelial cells and other glial cells 25. It should be noted that the strategic localization of GLUT2 in the apical membrane of tanycytes puts them in a privileged position to sense glucose variations in the CSF. It is possible that a low expression of GLUT2 in the hypothalamic nuclei exists, which has prevented researchers from obtaining conclusive results *via* immunocytochemistry regarding the expression or localization of GLUT2 in GE or GI neurons.

Studies performed in ripglut1; glut2−/− mice, showed that lateral i.c.v. injection of glucose to mimic hyperglycaemia decreased NPY and increased POMC mRNA levels, which correlated with the cessation of FI 63. In experiments using the same mice, stimulated glucagon secretion was restored with the expression of GLUT2 by glial cells but not neurons, indicating the importance of glial cells in the central regulation of glucagon secretion 130. Furthermore, selective destruction of tanycytes through III-V injection of alloxan, a GK inhibitor and toxin that enters cells through GLUT2, inhibits the counter-regulatory responses generated by hypoglycaemia without damaging neurons in the AN, which again supports the involvement of tanycytes in the glucose sensing mechanism 131. Thus, in morphological and molecular terms, it is feasible to propose that tanycytes are functionally and metabolically coupled with hypothalamic neurons that participate in the regulation of FI.

## Participation of GK in hypothalamic glucosensing

Cerebral glucose metabolism is limited by the capacity to capture it *via* GLUTs and incorporate it into the glycolytic pathway through hexokinase phosphorylation 116,117. An elevated Km for glucose transport and the presence of GK (HK IV) imply cells could increase their glucose uptake rate in direct proportion to extracellular changes in glucose concentration. This property of GLUT2 and GK determines their participation in the glucose sensing mechanism of pancreatic β cells 45,132–135. Glucokinase catalyses the phosphorylation of glucose to glucose-6-phosphate with low affinity (S_0.5_ 5–15 mM) and is not inhibited by its product under physiological conditions 125,136. Glucokinase is a product of one gene; an alternative promoter is used in hepatic and pancreatic tissues, generating tissue-specific isoforms that differ in the first 15 amino acids 137. In the hypothalamus, RT-PCR and *in situ* hybridization analyses have revealed the expression of the pancreatic isoform of GK 45,136,138, which was confirmed by immunoblotting and enzyme assays 66,125,136,139–141. However, the expression of non-functional isoforms of GK produced by alternative splicing has been described in the hypothalamus and pituitary 136,142,143. Western blot and immunohistochemistry analyses in adult rats have shown the nuclear localization of GK in β1-tanycytes in the euglycaemic condition, as well as its expression by a small proportion of periventricular neurons 66. However, in early development GK mRNA levels were strongly up-regulated during the second post-natal week 144 and, GK was localized in the cytoplasm of tanycytes but not in the nucleus 66. Interestingly, at the same stage a similar subcellular distribution has been observed in hepatocytes 145. Hepatic GK activity is regulated at the post-translational level through interaction with GKRP, which functions as an anchor protein, modulating GK activity and mediating its nuclear translocation 146,147. Therefore, the data previously described suggest that nuclear compartmentalization of GK in tanycytes may be associated with post-natal GKRP co-expression, which may regulate GK activity in tanycytes in accordance with the metabolic needs of the cell.

In the hypothalamus, isotopic *in situ* hybridization revealed GKRP expression in the PVN as well as in periventricular glial cells 148. Recombinant proteins obtained by cloning GKRP from highly enriched primary tanycyte cultures have very high sequence identity with hepatic GKRP 24. However, different reports call into question if hepatic GKRP can regulate the activity of pancreatic GK 148–150, especially given that some studies failed to observe GKRP expression in the pancreas 149,150 with the exception of an alternatively spliced GKRP variant expressed in β cells 148. Recently, we performed a comparative study of GK distribution in response to different glycaemic conditions in the hypothalamus and liver. In the hypothalamus, increased GK nuclear localization was observed in hyperglycaemic conditions; however, it was primarily localized in the cytoplasm in hepatic tissue under the same conditions 24. Different reports have demonstrated that in liver GK interacts with GKRP in the nucleus in an inactive state, in hypoglycaemia 145,151–153. Using primary cultures of tanycytes the nuclear localization of GK and GKRP increased in the presence of high glucose concentration, which confirmed the *in situ* results. Supporting these results, it has been recently demonstrated that GK activity in the hypothalamus, and not in other cerebral regions, is increased with fasting 154. Thus, in tanycytes, the GK/GKRP complex can act as a molecular switch to arrest cellular responses to increased glucose.

## Conclusions

We have described the role of metabolic coupling between tanycytes and neurons in hypothalamic glucosensing, control of feeding behaviour and peripheral glucose homeostasis. The role of tanycytes in sensing glucose concentration in the CSF is illustrated by (*i*) the expression of GLUT1 and GLUT2 in the membrane that makes contact with the CSF, (*ii*) the expression of GK and GKRP, (*iii*) the evidence that tanycytes produce ATP-mediated Ca^2+^ waves in response to increases in extracellular glucose concentration and (*iv*) the data showing that tanycytes release lactate using MCT1 and MCT4. Our recent data show that orexigenic and anorexigenic neurons of the AN highly express the MCT2 isoform involved in monocarboxylate uptake. Thus, tanycytes are likely metabolically coupled with neurons of the hypothalamus *via* monocarboxylates, where lactate acts as an intercellular signalling molecule. Taken together, the possible role of glia, and in particular tanycytes, in regulating feeding behaviour in the hypothalamus has largely been underestimated. Further studies to better explore this regulatory system will allow identifying the precise deficiencies that are responsible for deregulation of these circuits in common diseases, such as diabetes and obesity. Finally, *in vivo* studies are necessary to demonstrate that the tanycyte-neuron interaction is required for hypothalamic glucosensing.
